# Intracellular Protective Functions and Therapeutical Potential of Trehalose

**DOI:** 10.3390/molecules29092088

**Published:** 2024-05-01

**Authors:** Dorota Kuczyńska-Wiśnik, Karolina Stojowska-Swędrzyńska, Ewa Laskowska

**Affiliations:** Department of General and Medical Biochemistry, Faculty of Biology, University of Gdansk, Wita Stwosza 59, 80-308 Gdansk, Poland; dorota.kuczynska-wisnik@ug.edu.pl (D.K.-W.); karolina.stojowska-swedrzynska@ug.edu.pl (K.S.-S.)

**Keywords:** trehalose, protein stability, free radical scavenger, bacterial pathogens

## Abstract

Trehalose is a naturally occurring, non-reducing saccharide widely distributed in nature. Over the years, research on trehalose has revealed that this initially thought simple storage molecule is a multifunctional and multitasking compound protecting cells against various stress factors. This review presents data on the role of trehalose in maintaining cellular homeostasis under stress conditions and in the virulence of bacteria and fungi. Numerous studies have demonstrated that trehalose acts in the cell as an osmoprotectant, chemical chaperone, free radical scavenger, carbon source, virulence factor, and metabolic regulator. The increasingly researched medical and therapeutic applications of trehalose are also discussed.

## 1. Introduction

In the environment, organisms rarely thrive under circumstances that are not ideal for growth; therefore, they have developed several mechanisms to survive in adverse conditions, such as thermal stress, salt and oxidative stress, desiccation, UV radiation, and exposure to heavy metal ions or pathogens. Among the many mechanisms prokaryotic and eukaryotic organisms have developed, low-molecular-weight organic osmolytes play a significant role. They are osmotically active compounds that can be divided into two main groups: (1) sugar and polyols (e.g., glycerol, sorbitol, and trehalose) and (2) amino acids and their derivatives (glycine betaine, proline, and glutamate). The primary role of osmolytes is to protect cells against osmotic stress. They are also highly effective in the response of organisms to various environmental stresses affecting the stability and structure of proteins and lipids [1,2]. Various osmolytes, including sugars and amino acids, can safely accumulate in cells without affecting cellular functions [3]. Among natural osmolytes, trehalose is a particularly interesting, versatile molecule with diverse roles in many organisms. The Web of Science database contains over 16,000 original scientific papers on trehalose; particular research interest is focused on its role in the pathogenesis of bacterial and fungal infections.

Trehalose (C_12_H_22_O_11_, α-D-glucopyranosyl-[1,1]-α-D-glucopyranoside) is a disaccharide comprised of two glucose monomers linked by α, α-1,1-glycoside bond. Thermodynamically and kinetically, it is the most stable disaccharide in nature [4]. Trehalose was first isolated in 1832 from rye ergot [5] and is now known to be produced by various organisms, including bacteria, fungi, invertebrates, and plants. In yeast and fungi, trehalose is produced in spores, fruiting bodies, and vegetative cells. Only vertebrates cannot synthesize trehalose but can use it as an exogenous carbon source since they have trehalase, the enzyme required to hydrolyze trehalose. In humans, trehalase occurs in the cells of the epithelial membrane of the small intestine and the kidneys [1,5].

Trehalose is a unique type of bioprotectant with exceptional properties, including high hydrophilicity, chemical stability, the ability to form nonhygroscopic glass, lack of internal hydrogen bonds, and strong resistance to both acid hydrolysis and cleavage by glucosidases. These physical and chemical properties of trehalose arise from its non-reducing properties. Besides, trehalose can occur in a cell in several polymorphs, from the crystalline (as commonly occurring trehalose dihydrate form) to amorphous states [4].

## 2. Mechanisms Governing the Accumulation of Trehalose

The level of trehalose varies significantly depending on the age of the cells, stage of growth, nutritional state and environmental conditions. For example, in insects, trehalose is used as an energy source, constituting 80–90% of the overall sugar content in the hemolymph. During the prepupal period, trehalose is intensely exploited for energy production necessary for metamorphosis [6]. In fungi spores, trehalose may constitute up to 10% of dry-weight basis (as in ascospores of *Neurospora tetrasperma*) and is rapidly consumed during germination [7]. *Saccharomyces cerevisiae* cells accumulate trehalose in the stationary phase, during temperature shifts from 30 to 45 °C and in the presence of other agents that induce heat shock response (ethanol, copper sulphate, or hydrogen peroxide) [8,9]. In commercial baker’s yeast, trehalose may constitute up to 20% of cell dry weight [10].

**Figure 1:**
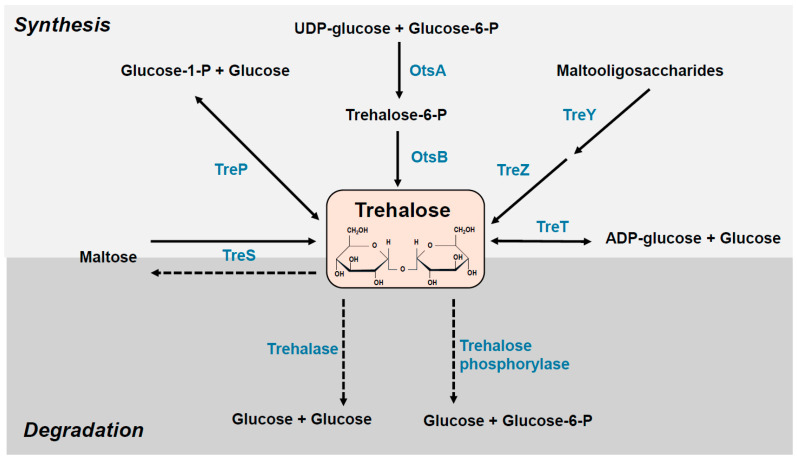
Trehalose synthesis and degradation pathways. See the text for more details. OtsA, trehalose-6-phosphate synthase; OtsB, trehalose-6-phosphate phosphatase; TreP trehalose phosphorylase; TreS, trehalose synthase; TreT, trehalose glycosyltransferase; TreY, maltooligosyl trehalose synthase; TreZ, maltooligosyl trehalose trehalohydrolase.

The levels of intracellular trehalose and other osmoprotectants depend on a well-controlled balance between enzymatic synthesis, uptake, and degradation. To use exogenous trehalose as a carbon source, enzymes capable of its degradation are necessary (Figure 1). Trehalose is hydrolyzed by two enzymes: trehalose phosphorylase and trehalase. The first is responsible for converting trehalose to trehalose-6-phosphate, which is then hydrolyzed to glucose and glucose-6-phosphate. Trehalase (α-glucosidase), an enzyme in many organisms, including mammals, hydrolyses trehalose to glucose. Two types of trehalases are classified according to their pH optima: cytoplasmic, neutral trehalase and extracellular acid trehalase [11]. In *E. coli*, two trehalose degradation pathways are known: periplasmic TreA trehalase, induced at high osmolarity, and cytoplasmic TreF, responsible for degrading trehalose synthesized in the cytoplasm [12].

Several pathways for trehalose synthesis have been identified (Figure 1). In the two-step pathway OtsAB (TPS/TPP), trehalose synthesis is catalyzed by trehalose-6-phosphate synthase (OtsA) and trehalose-6-phosphate phosphatase (OtsB). In the first reaction, glucose 6-phosphate and UDP-glucose are converted into trehalose 6-phosphate by OtsA. Subsequently, trehalose-6-phosphate is dephosphorylated and converted into trehalose by OtsB. The OtsAB pathway is the most common route in prokaryotes and eukaryotes. In *Escherichia coli*, the *otsA* and *otsB* genes form one operon controlled by RpoS, a specialized sigma S factor and a conserved stress regulator. The *otsAB* expression is induced by entry into the stationary phase, osmotic shock, extreme heat, cold and desiccation [13,14]. The *otsAB* genes participate in sigma S-dependent stationary-phase thermotolerance but are dispensable for adaptive thermotolerance in growing cells [15]. In the TreS pathway, trehalose is directly converted from maltose by trehalose synthase TreS, which catalyzes the reverse reaction–the synthesis of maltose from trehalose. In the TreYZ pathway, trehalose is synthesized from linear α-glucans polymer in two steps catalyzed by maltooligosyl trehalose synthase (TreY) and maltooligosyl trehalose trehalohydrolase (TreZ). The TreS and TreYZ pathways have been found only in prokaryotes. There are two other trehalose synthesis pathways: TreP (described in fungi and the protist *Euglena*) and TreT (found in bacteria and archaea). Trehalose phosphorylase (TreP) mediates the reversible formation of trehalose from glucose-1-phosphate and glucose, while trehalose glycosyltransferase, TreT, catalyzes the formation of trehalose from ADP-glucose and glucose [14,15,16,17,18]. Different trehalose synthesis pathways may co-occur in some organisms. For example, *Mycobacterium tuberculosis* and *Corynebacterium glutamicum* produce trehalose using the OtsA/B, TreY/Z and TreS pathways [19,20].

## 3. Intracellular Protective Functions of Trehalose

### 3.1. Trehalose as an Osmoprotectant and Chemical Chaperone

Trehalose plays a vital role as a stress protectant during major abiotic stresses such as dehydration and rehydration, high or low temperature, chemical toxicity and oxidative stress. The role of trehalose in protecting cells against the effects of desiccation is particularly well documented [21,22,23]. The accumulation of trehalose and other soluble sugars is a common feature of many desiccation-tolerant organisms, which are known as anhydrobiotes. This group of organisms includes yeast, some microorganisms, plants, and invertebrates [21,24,25]. Water loss imposes several stresses, including hyperosmolarity, hyper oxidation and hyperionicity, resulting in the concentration of solutes (sugars, salts, amino acids) and changes in the ionic strength and pH of the intracellular solution. Reduction of the hydration shell around the protein leads to conformational changes, resulting in a loss of enzymatic activity, denaturation, and aggregation. In the case of nucleic acid damage caused by dehydration, chemical modifications such as alkylation or oxidation occur. During water loss, membranes become disrupted, leading to the loss of compartmentation [22,26]. Trehalose protection against such lethal damages in desiccation-tolerant cells may involve different mechanisms: water replacement activity, water entrapment, and vitrification (glass formation). Trehalose replaces water by forming hydrogen bonds with polar residues of lipid and protein molecules, which prevents the aggregation of biomolecules and the disintegration of membranes. According to the water entrapment (water layer) hypothesis, trehalose forms a coating layer around proteins; the water is trapped in the intermediate layer between trehalose and the protein surface. The vitrification (or mechanical entrapment) theory suggests that trehalose accumulation leads to the formation of stable glasses, which may hold biomolecules in appropriate conformation and reduce the rates of chemical reactions [27,28].

Multiple studies have reported that trehalose and other osmolytes (betaine, proline, or glycerol) can function as chemical chaperones that protect proteins against the loss of activity, prevent chemical and thermal denaturation, and assist in the refolding of unfolded polypeptides [29]. Due to its exceptional stability, trehalose was found to be a better chaperone under highly stressful conditions, such as desiccation, than protein chaperones [30]. Regardless of the exact mechanism of action (water replacement, water entrapment, or vitrification), the extraordinary ability of trehalose to change the aqueous environment surrounding the protein and to preserve its native conformation makes trehalose the best protein stabilizer. It must be noted that although trehalose has been found to inhibit protein aggregation, its higher concentrations may “over-stabilize” proteins in a rigid conformation, leading to enhanced protein precipitation and aggregation [31,32,33].

### 3.2. Trehalose as a Free Radicals Scavenger

Multiple studies have demonstrated that trehalose protects cells from the detrimental effects of oxidative stress as a free radicals scavenger [34,35,36,37,38]. Reactive oxygen species (ROS) comprise free radicals, such as superoxide anion radical (O_2_^−^) and hydroxyl radical (OH^·^) and non-radical species, such as hydrogen peroxide (H_2_O_2_), hypochlorous acid (HOCl), and singlet oxygen (^1^O_2_). O_2_^−^ and H_2_O_2_ are generated as byproducts of aerobic metabolism and are present at low, non-toxic cell concentrations. In defense against ROS, organisms use two main strategies: (1) antioxidant enzymes (superoxide dismutase, SOD; catalase, CAT; and glutathione-dependent enzymes) and (2) small antioxidant molecules (glutathione, uric acid, and vitamin C and E), which neutralize or prevent the appearance of ROS [39]. Under stress conditions, the antioxidant mechanisms could be inactive, presumably due to dysfunction of specific enzymes, and as a consequence, free radicals accumulate, causing damage to DNA, proteins, and lipids. In proteins, oxidative damage leads to the formation of carbonyl groups, which promote protein aggregation. It has been shown that trehalose can alleviate oxidative stress by reducing H_2_O_2_-induced ROS accumulation and preserving the activity of the main antioxidant enzymes, superoxide dismutase, in wheat leaves [34]. During oxidative stress, trehalose acts primarily as a scavenger of free radicals, thus blocking their interaction with proteins and lipids. The role of trehalose in protecting cells against oxygen radicals has been well documented in bacteria, yeast, and other fungi [34,35,36,40,41,42]. In *S. cerevisiae,* intracellular trehalose markedly increases viability upon exposure to oxidative stress and this protective role of trehalose is correlated with a reduction in lipid peroxidation [36,42]. It was found that *E. coli* Δ*otsA* cells that are unable to synthesize trehalose experience oxidative stress during heat shock and in the stationary phase; the main target of oxidative damage is unsaturated fatty acids present in membranes [40]. An increase in trehalose content upon a mild heat shock or exposure to a proteasome inhibitor improves the resistance of *S. cerevisiae* to oxygen radicals generated by a system composed of H_2_O_2_ and iron (III) chloride (FeCl_3_). In contrast_,_ impaired trehalose synthesis reduces yeast resistance to H_2_O_2_ [35]. Antioxidant trehalose activity in plants has been reported by Rohman et al. [43]. It was shown that trehalose improves the growth of maize seedlings by reducing the levels of Na^+^/K^+^ and ROS, thereby protecting maize plants from salt stress and phosphorus deficiency [43].

### 3.3. Regulatory Functions of Trehalose

It has long been suggested that the protective function of trehalose cannot be reduced to the role of an osmoprotectant or a chemical chaperone. Numerous studies have indicated that trehalose and the products of its metabolism have important regulatory functions. For example, it was shown that in plants, trehalose-6-phosphate is involved in the regulation of glucose metabolism and plant development [17]. Blazquez et al. (1993) demonstrated that in *S. cerevisiae*, some components of the trehalose metabolism pathway may have other functions beyond trehalose production. It was found that trehalose-6-phosphate competitively inhibited the hexokinase, leading to altered glucose metabolism [44]. Consequently, loss of Tps1 (trehalose-6-phosphate synthase) broke down *S. cerevisiae* growth in the presence of glucose, most likely due to increased glucose flux into glycolysis [44]. In *S. cerevisiae* strains with a deletion in the *tps1* gene, the mutation complementation by *E. coli otsA* resulted in wild-type trehalose levels but only partially restored high-temperature survival and sporulation. Therefore, it has been suggested that not the accumulation of trehalose but the enzymes participating in its synthesis pathway are responsible for the stress resistance effect [45,46,47]. In the plant pathogenic fungus *Magnaporthe grisea*, Tps1 regulates the pentose phosphate pathway, thus affecting NADPH’s intracellular levels and integrating carbon and nitrogen metabolism [48,49].

We have shown recently that in *E.coli*, trehalose protects proteins against aggregation during the stationary phase by acting not only as a chemical chaperone but also as a metabolite [50]. Due to the lack of trehalose synthesis in Δ*otsA* cells, the excess of glucose-6-phosphate is converted to pyruvate and then to acetyl-CoA and acetyl phosphate (AcP). AcP is the main donor of acetyl groups for N^ε^-lysine acetylation in bacteria. Therefore, N^ε^-lysine acetylation was enhanced in Δ*otsA* cells. N^ε^-lysine acetylation is a reversible modification that significantly impacts the structure and function of proteins, which may result in increased protein aggregation [50,51,52,53]. Therefore, the lack of trehalose synthesis causes carbon overflow due to the accumulation of reactive acyl-CoA species, resulting in enhanced protein acetylation and aggregation. In conclusion, [50,51,52,53] this study demonstrated that trehalose may participate in maintaining proteostasis as a metabolite that indirectly counteracts protein acetylation and aggregation [50,54].

### 3.4. Trehalose and Neuroprotection

Trehalose has been reported to have neuroprotective functions in vitro and in animal models of various neurodegenerative diseases by inhibiting the aggregation of proteins involved in Huntington’s, Alzheimer’s, Parkinson’s, and prion diseases [55,56,57,58]. Tanaka et al. (2004) demonstrated that in vitro trehalose binding to expanded polyglutamines stabilizes the partially unfolded polyglutamine-containing protein and inhibits polyglutamine-mediated protein aggregation [56]. Additionally, orally administrated trehalose acts analogously in a transgenic mouse model of Huntington’s disease. Trehalose also effectively inhibits the aggregation of β-amyloid and reduces its neurotoxicity in human neuroblastoma cells [57]. The neuroprotective effects of trehalose may result from the direct protection of proteins prone to aggregation and may be associated with the activation of autophagy—one of the mechanisms responsible for the removal of aggregates [59,60,61,62]. Other studies suggest that trehalose does not function as an autophagy inducer but instead blocks the degradation of autophagosomes by lysosomes [63,64]. Therefore, the exact mechanism of neuroprotection by trehalose remains to be elucidated. Trehalose may pass through the blood–brain barrier and directly affects neuronal cells. However, it must be remembered that vertebrates do not synthesize trehalose, and trehalose administered in the diet is digested in the intestines to glucose. Therefore, it is possible that the neuroprotective effect of trehalose is indirect and may result from its antioxidant and anti-inflammatory properties, which beneficially affect gut microbiota [63,64,65]. An increasing body of evidence suggests that trehalose may contribute to the integrity of the microbiota–gut–brain axis by protecting the gut microbiota against detrimental stress factors [66]. For example, Buckley et al. demonstrated that trehalose remodels human microbiota in gut models, reducing *Clostridium difficile* infection and promoting the growth of competitive bacterial species [67]. One of these species, *Faecalibacterium prausnitzii*, produces short-chain fatty acids, butyrate and formate, which have been reported to regulate genes involved in microglia maturation [68]. In general, microorganisms can synthesize neuroactive compounds, including γ-aminobutyric acid and short-chain fatty acids, or induce host-derived production of neurotransmitters (oxytocin, brain-derived neurotrophic factor) to mediate gut–brain signaling. Bidirectional communication between the gut microbiota and brain includes the immune system and the vagus nerve [68,69]. It is known that microorganism-dependent signaling may affect social behavior, neuronal plasticity, and stress responsiveness in animal models [68].

### 3.5. Trehalose and Autophagy

**Figure 2:**
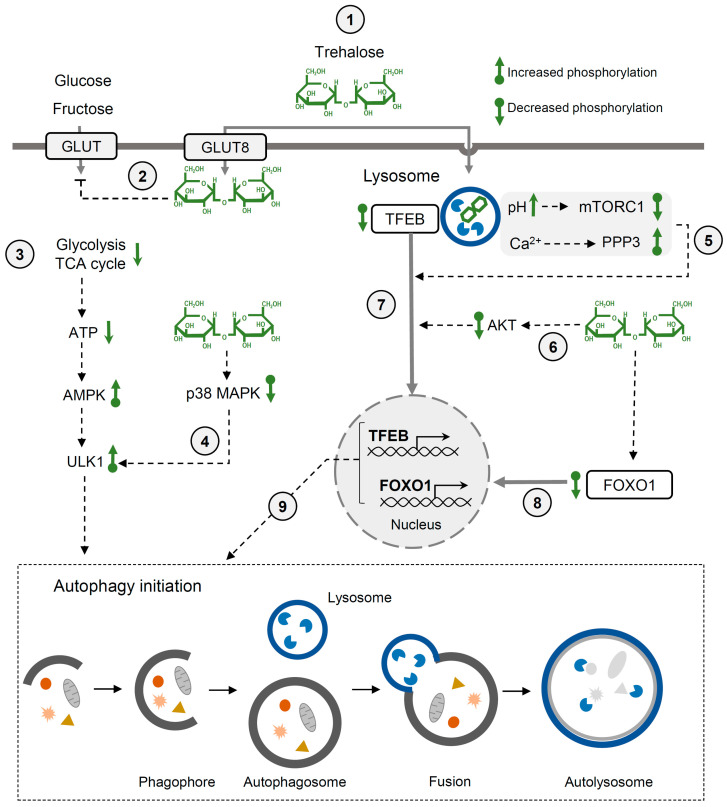
Regulation of autophagy by trehalose. (1) Transport of trehalose into mammalian cells occurs via GLUT8 (SLC2A8) transporter or endocytosis (2) Trehalose blocks the entry of glucose and fructose into the cell, which limits the amount of ATP produced by glycolysis and citric acid cycle (3). This leads to the starvation-like state that activates AMPK and ULK-1, initiating autophagy. (4) ULK-1 can also be activated by trehalose through the p38MAPK pathway. (5) Trehalose transported by endocytosis accumulates in lysosomes. This results in an increase in the lysosomal pH, which in turn leads to the inactivation of mTORC1. Another mechanism includes lysosomal membrane permeabilization, Ca^2+^ release, and PPP3 activation. Lysosomal dysfunction (5) and ATK inactivation by trehalose (6) can result in the dephosphorylation and translocation of the transcription factor TFEB to the nucleus (7). (8) Additionally, trehalose-dependent dephosphorylation of FOXO-1 can promote FOXO-1 translocation to the nucleus. (9) TFEB and FOXO1 can then activate the transcription of genes involved in autophagosome and lysosomal biogenesis. See the text for more details. The bottom panel illustrates the main phases of macroautophagy, the best-studied type of autophagy.

Multiple studies have demonstrated that trehalose induces autophagy and thereby reduces pathological hallmarks of various diseases, including protein aggregation diseases, cardiac injuries, diabetes, and viral infections [59,60,63,70,71]. Trehalose may affect autophagy in various cell types through different mechanisms (Figure 2). It has been demonstrated that trehalose may block the entry of glucose and fructose via GLUT transporters into hepatocytes [72]. Thereby, trehalose indirectly inhibits glycolysis and the citric acid cycle, leading to ATP deficiency. This low-energy state results in the activation of AMPK (AMP-activated protein kinase) and ULK1 (unc-51-like kinase 1) by phosphorylation, which triggers autophagy [72]. Increased levels of phosphorylated AMPK and ULK1 have been detected in mice liver after oral administration of trehalose. Another trehalose-dependent pathway detected in mice motoneurons is initiated after the transient enlargement of lysosomes and the permeabilization of lysosomal membranes upon osmotic stress caused by trehalose. Ca^2+^ ions released from dysfunctional lysosomes activate PPP3CB (the calcium-dependent, calmodulin-stimulated protein phosphatase), which dephosphorylates transcription factor EB (TFEB), enabling its translocation to the nucleus [60]. TFEB stimulates the expression of genes involved in autophagosome formation, autophagosome–lysosome fusion and lysosomal biogenesis. Recently, Jeong et al. showed that in macrophages, trehalose is taken up via endocytosis and accumulates in lysosomes. Trehalose retention in lysosomes impairs mild stress by increasing lysosomal pH [73]. This, in turn, inactivates MTOR1 (mechanistic target of rapamycin kinase) complex 1—the main TFEB regulator. Under non-stress conditions, active MTOR1 phosphorylates TFEB and other targets. In the presence of trehalose, the inactivation of MTOR1 leads to the nuclear translocation of TFEB and autophagy induction. Trehalose may also promote TFEB translocation by decreasing Akt kinase activity that phosphorylates TFEB. This trehalose-dependent regulation of the Akt-TFEB pathway has been demonstrated using HeLa cells, mice cortical neurons, and patient-derived JNCL (juvenile neuronal ceroid lipofuscinosis) fibroblasts characterized by the accumulation of ceroid lipopigment. It was also shown that the administration of trehalose to a mouse model of JNCL increased the clearance of ceroid lipopigment deposits and reduced neuropathy [62]. Trehalose may also activate autophagy through the dephosphorylation of FOXO1 (Forkhead box O) and p38 MAPK (p38 mitogen-activated protein kinase) [74]. This mechanism has been revealed while investigating the impact of trehalose on myocardial function in a mouse model of insulin resistance caused by Akt2 knockout. It was found that trehalose treatment significantly ameliorated Akt2 knockout-induced myocardial contractile dysfunction [74].

The induction of autophagy by trehalose may also affect the outcome of infections caused by herpesviruses, such as human cytomegalovirus (HMCV) and varicella-zoster virus (VZV) [75,76]. Belzile et al. demonstrated that trehalose facilitated autophagy in different human cell lines (foreskin fibroblasts, aortic endothelial cells, and neural stem cells) infected with HMCV, which resulted in decreased HMCV replication, gene expression, and cytopathic effects [75]. The effects of trehalose on VZV infection depended on the timing of trehalose addition and the type of inoculum used (cell-free virus or infected cells). Reduced VZV spread was observed only when cell monolayers pretreated with trehalose were infected with cell-free virus [76]. Trehalose has also been shown to inhibit infection of macrophages and CD4 T cells with human immunodeficiency virus type 1 (HIV) by inducing autophagy, which degraded intracellular viruses [77]. In addition, trehalose reduced virus entry by downregulating CD4 expression in macrophages and both CD4 and CCR5 expression in T cells.

### 3.6. Trehalose and Glucose Homeostasis

Several studies have suggested that trehalose may affect glucose metabolism and help maintain glucose homeostasis in diabetic patients. The molecular mechanisms underlying the glucose homeostasis regulated by trehalose have been discussed in detail in recent reviews [65,78]. Trehalose may impact glucose homeostasis through different pathways that contribute to insulin sensitivity and secretion, glucose and lipid metabolism, islet function, oxidative stress management, and inflammation [78]. In diabetes, prolonged hyperglycemia results in oxidative stress that triggers the expression of an adipokine MCP-1 (monocyte chemoattractant protein–1). Increased levels of MCP1 may promote insulin resistance and infiltration of macrophages into adipose tissue. A study by Arai et al. has shown that administering trehalose to obese mice fed a high-fat diet decreased insulin secretion and MCP-1 expression [79]. This, in turn, suppressed adipocyte hypertrophy and reduced insulin resistance. Further studies revealed that trehalose administration to obese mice mitigates insulin resistance by increasing the serum level of a high-molecular-weight isoform of adiponectin [80]. Adiponectin, a cytokine secreted by adipocytes, improves insulin sensitivity and reduces inflammation in peripheral tissues by enhancing the expression of two crucial regulators for glucose metabolism: IRS-1 and IRS-2 (insulin receptor substrates 1 and 2) [81]. It is worth noting that MCP-1 has been linked to numerous other diseases, such as Parkinsonism, ischemic heart disease, tuberculosis, COVID-19, and rheumatoid arthritis [82]. Therefore, utilizing trehalose as an antioxidant protectant and autophagy inducer could potentially be an effective treatment strategy for numerous diseases. Another pathway related to glucose homeostasis and affected by trehalose involves eLOX3 and PPARγ [83]. eLOX3, encoded by the Aloxe3 gene, is the epidermal-type lipoxygenase activated during fasting, glucose withdrawal, or trehalose treatment. It has been demonstrated that eLOX3 overproduction in hepatocytes increased insulin sensitivity and reduced weight gain and hepatic steatosis in obese mouse models [83]. These eLOX3-dependent effects required the PPARγ signaling pathways. PPAR-γ is a transcription factor highly expressed in adipose tissues and involved in glucose and lipid homeostasis, insulin sensitivity, adipogenesis, inflammation, immune response, and apoptosis.

## 4. Trehalose and Pathogenicity

An increasing body of evidence indicates that trehalose plays a crucial role in the pathogen and symbiont colonization of host cells. It is primarily related to protecting the pathogen against the host’s defense, but some reports indicate that trehalose also constitutes a virulence factor. This is particularly evident in plant pathogens and symbionts. Trehalose synthesis and metabolism are required for plant infections by fungi, as described in several reports (summarised in Table 1). Deleting the TPS1 gene, encoding trehalose-6-phosphate synthase, in the rice blast fungus *Magnaporthe grisea* leads to the inhibition of trehalose synthesis and, consequently, greatly attenuated pathogenicity [48]. The lack of trehalase (in the Δ*nth1* mutant), resulting in impaired trehalose degradation, inhibits fungus proliferation in infected plants [84]. Because plants also synthesize trehalose, some pathogens can obtain trehalose from host cells and use it as a carbon source. Interestingly, *Phytophthora sojae*, a pathogen of soybean (*Glycine max*), directly stimulates trehalose biosynthesis by the host. *P. sojae* produces an effector that interacts with soybean trehalose-6-phosphate synthase 6 (GmTPS6), increasing its enzymatic activity and enhancing trehalose accumulation in soybean. Consistent with these results, the GmTPS6 gene knockdown was found to inhibit *P. sojae* infection [85].

In host–bacteria interactions, trehalose fulfils the role of a bioprotectant or virulence factor (Table 1). *Bradyrhizobium diazoefficiens*, a soil bacterium that infects the roots of legume plants, experiences osmotically stressful conditions during the early stages of host infection. The elevated cytoplasmic trehalose level, produced by the trehalose-6-phosphate pathway, is crucial to overcome infection stress [104]. Greater sensitivity to osmotic stress combined with lower pathogenicity has also been demonstrated for other pathogenic bacteria that could not synthesize trehalose [99,105,106,107,108,109]. In mycobacterial species such as *M. smegmatis* and *M. tuberculosis*, trehalose is essential for growth and virulence as a precursor of trehalose lipids, which are critical cell wall components. Among them, trehalose-6, 6′-dimycolate, also known as the cord factor, decreases bacterial cell permeability (especially for drugs) and blocks mycobacteria fusion with macrophages and lysosomes [110,111]. It was also shown that the cord factor imparts exceptional desiccation resistance to mycobacterial membranes [112]. 

There is evidence that not only trehalose itself but also enzymes involved in trehalose biosynthesis and hydrolysis pathways act as virulence factors. *Burkholderia pseudomallei* mutant Δ*treA*, which is defective in terms of trehalose degradation, was characterized by an increased tolerance to thermal stress but produced less biofilm and showed reduced virulence in *Galleria mellonella* larvae and in mice [94]. Similarly, *Candida parapsilosis atc1*Δ cells that lack acid trehalase displayed higher resistance to saline exposure, heat shock and severe oxidative stress but were attenuated in larvae of *Galleria mellonella* [113]. Further studies revealed that trehalase activity was necessary for *Candida* biofilm formation [114]. This reduced virulence of trehalase deficient strain may result from slower growth of mutant strain or increased bacterial clearance by the host immune system. However, other mechanisms may be responsible for the association between trehalases and virulence. In an extraintestinal pathogenic *E. coli* MT78 strain, which is responsible for urinary tract infections, the deletion of the *treA* gene and lack of periplasmic trehalase impaired type 1 fimbria production. Reduced production of type 1 fimbriae resulted in decreased cell invasion capacity and virulence in a mouse urinary tract infection model [95].

Trehalose not only plays a significant role in the colonization of host cells and helps pathogens cope with stress during development but is also involved in persistence—another bacterial strategy that helps pathogens survive various stressful conditions. Persisters are dormant or slow-growing bacteria that can survive prolonged treatment by bactericidal antibiotics [115,116,117,118,119]. Persisters may arise spontaneously in any culture, but stressful conditions such as starvation and oxidative and acid stress enhance their formation. Numerous studies have indicated that multiple mechanisms underlie persister formation, including the stringent response, reduced cell energy, toxin–antitoxin modules, and protein aggregation. Trehalose may also indirectly contribute to *E. coli* persister formation by affecting protein aggregation or extracellular indole production [31,40]. It was found that during the stationary phase, persister frequencies correlated with protein aggregates’ levels, which were dependent on the concentration of externally added trehalose. Low trehalose concentrations inhibited protein aggregation and persister formation, whereas high trehalose concentrations enhanced aggregation and persisters levels. Increased levels of persisters were also produced in the *E. coli* Δ*otsA* mutant, which lacks trehalose-6-phosphate synthase. The disruption of trehalose synthesis in *E. coli* led to oxidative stress, enhanced protein aggregation, and the overproduction of extracellular indole [40,51]. Indole, as a signal molecule, has been shown to stimulate persister formation [40,120]. However, the opposite effect of indole on persister frequencies was reported by other studies [121,122]. It was also shown that in *M. tuberculosis*, the formation of persister cells is associated with a TreS-dependent trehalose-catalytic shift. In *M. tuberculosis* persisters, trehalose is used to synthesize central carbon metabolism intermediates instead of cell surface glycolipids. The trehalose–catalytic shift maintains ATP and NADPH, and thereby alleviates the effect of bedaquiline (BDQ) [123]. BDQ is a new antibiotic applied as part of combination therapy in adults with pulmonary multidrug-resistant tuberculosis.

## 5. Applications of Trehalose

The bioprotective properties of trehalose determine the wide use of this saccharide in biotechnology, pharmaceutical, food, and cosmetic industries, as well as in medicine. Trehalose is a natural component of the human diet; it occurs naturally in bread, honey, mushrooms, wine, and beer. It is also a popular food additive that acts as a sweetener and bulking agent, protects proteins and lipids in food against degradation by oxidation, heating or cooling, and prevents the loss of aroma and nutritional properties. In the cosmetic industry, trehalose is used as a moisturizing agent to block unpleasant odors. Trehalose and synthetic trehalose analogs are highly effective and versatile stabilizers used to extend the shelf life of vaccines, antibodies, and enzymes and for cryopreservation of stem cells, organs, and tissues [124,125,126,127]. Trehalose has been used in the treatment of dry eye disease as it can stabilize the lipid layer and reduce the osmolarity of the tear film, which prevents evaporation. Additionally, trehalose has been shown to regulate the expression of genes responsible for tear secretion and enhance the survival of corneal epithelial cells [128,129,130].

Trehalose is also considered a potential new drug in the treatment of neurodegenerative diseases and other pathological conditions, as already mentioned in Section 3.4, Section 3.5 and Section 3.6. It has been demonstrated that systemic administration of a neurotherapeutic dose of trehalose is harmless, as it does not affect body weight, survival and morphology of the liver, pancreas or kidney [131]. In addition to neuroprotection, dietary trehalose may have other beneficial health effects [132,133,134]. It has been demonstrated that the addition of dietary trehalose, instead of glucose, prolongs lifespan and promotes healthspan in *C. elegans*. The same effect was promoted by the metabolic shift that inhibited glycogen synthesis from glucose (via inactivation of glycogen synthase) or limited trehalose degradation (due to the lack of trehalase). This anti-aging function of trehalose was dependent on the FOXO transcription factor DAF-16 and was associated with the upregulation of autophagy [134]. To date, the ClinicalTrials database (https://clinicaltrials.gov/, accessed on 20 April 2024) has recorded over 60 trials assessing the therapeutic potential of trehalose. These trials are at various stages, from recruitment to completed studies, and include treatments for Alzheimer’s disease, Parkinson’s disease, spinocerebellar ataxia type 3, amyotrophic lateral sclerosis, type 2 diabetes, cardiovascular disease, and dry eye disease.

Due to the involvement of trehalose in pathogenicity and the lack of a trehalose synthesis pathway in mammals, trehalose-metabolizing enzymes (particularly trehalose-6-phosphate synthase and trehalose-6-phosphate phosphatase) have been considered promising targets for antifungal or antibacterial therapies. Trehalose-6-phosphate phosphatase, in particular, seems to be a promising target of antifungal therapies, as it is very specific and impacts the virulence and pathogenesis of many fungi [135]. Furthermore, the enzymes responsible for the degradation of trehalose significantly impact virulence and are, therefore, potential therapeutic targets. For example, structural analogs of trehalose, such as validamycin A, which acts as a competitive inhibitor of acid trehalases, have been successfully used since the 1980s to protect plants against fungal infections [136]. Validamycin A inhibits several plant and insect trehalases equally strongly but is less effective against human pathogens (fungi and bacteria). However, new degradation-resistant trehalose analogs that could eradicate human pathogens are being sought [137]. Some trehalose analogs bearing modifications at the 6-position, such as 6-azido-6-deoxy-α, and α′-trehalose, inhibit growth and biofilm formation in *Mycobacterium smegmatis* [138]. 6-azido-6-deoxy-α,α′-trehalose competitively inhibits TreS, sensitizing drug-tolerant *M. tuberculosis* persisters to BDQ [123]. It has also been reported that the efficiency of ampicillin and kanamycine in killing *E. coli* persisters was improved in the presence of trehalose [31]. Thus, trehalose and its homologs may represent an interesting example of new drugs for the development of adjunctive therapies.

The potential role of trehalose in protecting plants against pathogens is also being investigated. It has been shown, for example, that the exogenous use of trehalose increases the protection of *Arabidopsis thaliana* against peach aphid or enhances the resistance of wheat (*Triticum aestivum* L.) to powdery mildew. Additionally, pearl millet seeds treated with trehalose before sowing showed approximately 70% protection against downy mildew in greenhouse conditions. Importantly, the protective effect of exogenous trehalose is based on the induction of protective mechanisms in the host and does not affect the formation of spores in the pathogen. Therefore, trehalose may be a promising and safe alternative to pesticides [139].

## 6. Conclusions

The continuing interest in trehalose translates into a constantly growing number of reports on its role in various processes occurring in living organisms. It is known that trehalose provides protection against the effects of abiotic stress experienced by organisms in the environment. At the molecular level, trehalose protects and stabilizes macromolecules, acting as a free radical scavenger and chemical chaperone, which is more stable and effective in extreme conditions than protein chaperones. The role of trehalose as a signaling molecule involved in the regulation of metabolism in microorganisms still remains unclear, and trehalose targets, as well as mechanisms of its action, should be identified. There is increasing evidence indicating that trehalose plays a significant role in promoting autophagy, which may help to develop effective therapeutic strategies for treating neurodegenerative diseases, diabetes and other diseases. However, it is important to note that dietary trehalose is digested in the intestines, meaning that the most beneficial health effects of trehalose may be indirect. All these issues require further, in-depth research.

Trehalose has gained more attention since it has been found to be involved in the virulence of pathogens. On the one hand, it allows pathogens to penetrate and develop in host cells; on the other hand, it activates mechanisms in the host cells that combat infections. The role of trehalose in pathogenicity is still not fully understood, but the trehalose metabolism pathway seems to be a promising target for new antibacterial and antifungal therapies. The latest reports on the role of trehalose in bacterial persistence indicate the possibility of its use in combined approaches against infections, which reduces the failure of antimicrobial therapy. 

## Figures and Tables

**Table 1:** Influence of mutations in trehalose synthesis and degradation pathways on the virulence and survival of fungi and bacteria under abiotic stress conditions.

Species	Mutation	Virulence	Survival under Abiotic Stress Conditions	Ref.
**Fungi**				
*Aspergillus fumigatus*	*tpsA tpsB* (trehalose-6-phosphate synthase genes)	Hypervirulent in a murine model of invasive aspergillosis	Delayed germination at 37 °C, reduced thermotolerance at 50 °C and increased susceptibility to oxidative shock	[86]
	*orlA* (trehalose-6P phosphatase)	Virulence attenuation in murine models of invasive pulmonary aspergillosis, increased sensitivity to cell wall perturbing agents (Calcofluor white, Congo Red or nikkomycin Z)		[87]
*Botrytis cinerea*	*tps1* (trehalose-6-phosphate synthase)	Similar to the wild-type strain	Normal vegetative growth; growth inhibition at higher temperature	[88]
	*tre1* (neutral trehalase)	Delayed germination in the presence of sugars at low concentrations	Increased heat resistance	
*Candida albicans*	*tps1*	Lower infection rate in mice; more sensitive to macrophage killing		[89]
	*tps2* (trehalose-6P phosphatase)	Reduces virulence; more susceptible to murine and human macrophages phagocytosis	Reduced growth at high temperatures, sensitivity to heat shock (42 °C) and oxidative exposure (50 mM H_2_O_2_)	[90]
*Cryptococcus neoformans*	*tps1* *tps2*	Avirulent in rabbits and mice	Temperature sensitivity at 37 °C in the presence of glucose	[91]
	*nth1* (neutral trehalase)	Similar to the wild-type strain		
*Fusarium graminearum*	*TPS2*	Reduction in mycotoxin production; 99% lower virulence on wheat		[92]
*Magnaporthe grisea*	*TPS*	Reduced sporulation and virulence		[84]
	*NTH1*	Decreased ability to colonize plant tissue		
*Verticillium dahliae*	*Vdpt1* (neutral trehalase)	Reduced colony growth rate, delayed mycelial growth and conidial germination and reduced pathogenicity	Increased resistance to high temperature, NaCl, sorbitol, and validamycin A	[93]
**Bacteria**				
*Burkholderia pseudomallei*	*treA* (trehalase)	Decreased biofilm formation; reduced virulence in *G. mellonella* and in mice; reduced growth in murine macrophages	Increased tolerance to thermal stress	[94]
*Escherichia coli (ExPEC)* strain MT78	*treA*	Reduces cell invasion and colonization of the bladder in a murine urinary tract infection model; reduced production of type 1 fimbriae	Increased resistance to 0.6 M urea	[95]
*Escherichia coli*	*otsA* (trehalose-6-phosphate synthase)	Increased formation of antibiotic (Amp and Ofx) tolerance cells after heat shock and in stationary cultures	Reduced ability to survive at 4 °C Increased oxidative stress damage	[14,40]
	*otsA otsB* double mutation		Decreased stationary phase thermotolerance at 55 °C	[15]
*Klebsiella pneumoniae* NTUH-K2044	*treC* (trehalose-6-phosphate hydrolase)	Decreased capsule production; decreased biofilm formation; attenuated ability to colonize the gastrointestinal tract in a murine model		[96]
*Listeria monocytogenes*	*treA* (phosphotrehalase)		Enhanced resistance to heat, high osmolarity, desiccation, and freeze–thaw cycling stresses.	[97]
*Mycobacterium tuberculosis*	*otsB2* (trehalose-6-phosphate phosphatase)	Lethal		[98]
	*otsA*	Growth defect during in vitro culture and murine infection		
	*treS* (trehalose synthase)	Reduced growth in the mouse model		
*Pseudomonas aeruginosa* strain PA14	*treYZ* (maltooligosyl trehalose synthase and maltooligosyl trehalose trehalohydrolase	Severe attenuation in virulence in *Arabidopsis* leaves		[99]
	*treS*	Attenuation in virulence in *Arabidopsis* leaves		
	double Δ*treYZ*Δ*treS* mutant	Severe attenuation in virulence in Arabidopsis leaves; full virulence in nematodes, insects, or mice		
*Ralstonia solanacearum*	*treA*	Defective in tomato colonization, reduced virulence		[100]
*Rhizobium leguminosarum* bv. *trifolii*	*otsA treY*	Less competitive for nodule occupancy than the wild-type strain	Decreased resistance to drying and subsequent storage at 25 °C	[101]
*Salmonella enterica* serovar Typhimurium	*otsA*	The ability to colonize spleen tissues in mice similar to the wild-type strain	Growth defect at 45 °C in minimal medium containing 0.2 M NaCl	[102,103]
